# Carboxylic acids derived from triacylglycerols that contribute to the increase in acid value during the thermal oxidation of oils

**DOI:** 10.1038/s41598-022-15627-3

**Published:** 2022-07-21

**Authors:** Masayoshi Sakaino, Takashi Sano, Shunji Kato, Naoki Shimizu, Junya Ito, Halida Rahmania, Jun Imagi, Kiyotaka Nakagawa

**Affiliations:** 1 Food Design Center, J-OIL MILLS, INC., Yokohama, Kanagawa 230-0053 Japan; 2 Laboratory of Food Function Analysis, Graduate School of Agricultural Science, Tohoku University, Sendai, Miyagi 980-8572 Japan; 3 J-Oil Mills Innovation Laboratory, Graduate School of Agricultural Science, Tohoku University, Sendai, Miyagi 980-8572 Japan

**Keywords:** Biochemistry, Lipids, Oils, Biological techniques, Mass spectrometry

## Abstract

Acid value (AV), is a widely used indicator of oil degradation that, by definition, measures the free fatty acids formed via the hydrolysis of triacyclglycerols. However, based on observations made in previous studies, we hypothesized that the oxidation of triacylglycerols leads to the formation of carboxylic acids with a glycerol backbone which are also calculated as AV. In this study, we aimed to identify such carboxylic acids and prove the above hypothesis. Heating a canola oil at 180 °C for 6 h without the addition of water resulted in an increase in AV from 0.054 to 0.241. However, the contribution of free fatty acids to this increase in AV was minimal; free fatty acid-derived AV before and after heating was 0.020 and 0.023, respectively. Then, via mass spectrometric analyses, we identified two 8-carboxy-octanoyl (azelaoyl) -triacylglycerols (i.e., dioleoyl-azelaoyl-glycerol and oleoyl-linoleoyl-azelaoyl-glycerol) in the heated oil. Azelaoyl-triacylglycerols-derived AV before and after heating the oil was 0.008 and 0.109, respectively, demonstrating that azelaoyl-triacylglycerols contribute to AV. Such an increase in AV by azelaoyl-triacylglycerols was also observed in an oil used to deep-fry potatoes (i.e., an oil with a relatively high water content). These results suggest that AV is also an indicator of the thermal oxidation of triacylglycerols.

## Introduction

Vegetable oils (e.g., canola, soybean, palm, and olive oil) are widely used in food applications. However, oil deterioration during food manufacturing, cooking, and storage can lead to undesirable flavor and taste of foods^1–3^. Thus, the assessment of oil deterioration is highly important to ensure the quality of food products^4^.

Under extensive food processing (e.g., deep-fry) and inadequate storage conditions (e.g., high temperature and humidity), triacylglycerols, the main constituent of oils, gradually hydrolyze into glycerol and free fatty acids^5^. Therefore, acid value (AV), which measures the free fatty acid content of oils, has been widely and frequently used as an indicator of oil deterioration. AV of an oil is determined by titration of carboxylic acids (RCOOH) with potassium hydroxide (KOH) as shown in the reaction below.

$${\text{KOH }} + {\text{ RCOOH }} \to {\text{ RCOOK }} + {\text{ H}}_{{2}} {\text{O}}$$

In ISO 660, AV is defined as the amount of potassium hydroxide (in milligrams) necessary to neutralize the free fatty acids contained in 1 g of sample^6^. Some countries such as Japan and Austria specify that frying oils with an AV above 2.5 should be discarded^7^.

**Figure 1:**
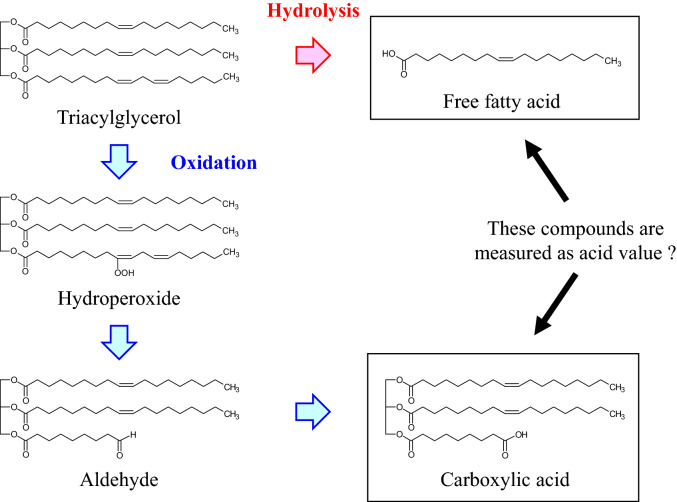
Hypothesis regarding the formation of carboxylic acids by the heating of canola oil.

Based on the principle above, an increase in AV can be rephrased as the “progress of triacylglycerol hydrolysis” in an oil (Fig. 1). Thus, by definition, water is required to increase the AV of an oil. Several studies, however, have observed an increase in AV by heating oils under conditions without the addition of water^8–13^. For example, Ogata *et al.* observed an increase in the AV of soybean oil by simply heating the oil at 180 °C^8^. However, the compounds that caused this increase in AV were not mentioned in the study. Meanwhile, Fujisaki *et al.* observed an increase in the AV of high-oleic safflower oil by heating the oil at 180 °C and argued that this was due to the formation of free fatty acids^9^. However, as we discuss in later sections, simply heating an oil without the addition of water presumably does not provide enough water to significantly hydrolyze triacylglycerols. Furthermore, in the same study, the increase in AV caused by heating was suppressed by lowering atmospheric oxygen concentrations. Hence, another possible explanation for the increase in AV is that the triacylglycerols in the oil were oxidized, leading to the formation of carboxylic acids which were calculated as AV. However, to the best of our knowledge, no study has verified that the oxidation of triacylglycerols induces an increase in AV. Moreover, compounds other than free fatty acids that contribute to AV have not been identified.

Based on the above background, we hypothesized that the oxidation of triacylglycerols leads to the formation of carboxylic acids with a glycerol backbone (Fig. 1), and these carboxylic acids can be calculated as AV. Hence, the aim of this study was to prove this hypothesis and to identify and quantitate the carboxylic acids that contribute to the heating-induced increase in AV. Consequently, we first confirmed that heating a vegetable oil (i.e., canola oil) without the addition of water results in a significant increase in AV. Then, carboxylic acids with a glycerol backbone that contributed to this increase in AV were identified by ultra-high performance liquid chromatography time-of-flight mass spectrometry (UPLC-Tof/MS). Quantification of the carboxylic acids was performed by gas chromatography-mass spectrometry (GC-MS). The identified carboxylic acids were also found in an oil used to deep-fry potatoes (i.e., an oil with a relatively high water content), suggesting their further contribution to AV. These results suggested that AV is not only an indicator of triacylglycerol hydrolysis but also an indicator of triacylglycerol oxidation. These findings should contribute to improving the quality of various oils and foods.

## Results and discussion

### Confirmation that heating an oil without the addition of water leads to an increase in AV

As described in the introduction, AV is an important indicator to evaluate the quality of vegetable oils. AV, by definition, measures the amount of free fatty acids formed by the hydrolysis of triacylglycerols. Nevertheless, several studies have observed an increase in AV by simply heating vegetable oils without the addition of water^8–13^. The reasons underlying this phenomenon have not been evaluated in previous studies. In this study, we hypothesized that the oxidation of triacylglycerols leads to the formation of carboxyl acids that possess a glycerol backbone (Fig. 1), and these carboxylic acids are calculated as AV.

Firstly, we aimed to confirm that the heating of a vegetable oil induces an increase in AV even when no water is added to the oil. Fresh canola oil (10 g) was heated in a 51 mm stainless steel dish at 180 °C for 6 h. Heating the oil caused a significant increase in AV from 0.054 to 0.241. Other parameters (color and viscosity) were also increased (Table 1). These findings were in good agreement with the results of previous studies^8–13^.

**Table 1:** Changes in AV, free fatty acid-derived AV, color, and viscosity by the heating of canola oil.

	AV	Free fatty acid- derived AV	Color (10R + Y)	Viscosity (mPa s)
Before heating	0.06 ± 0.00	0.020 ± 0.001	1.7 ± 0.0	46.8 ± 0.2
After heating	0.24 ± 0.03	0.023 ± 0.001	4.5 ± 0.2	58.1 ± 1.2

Mean ± SD.

Fresh canola oil was placed in a stainless steel dish and heated on a digital heat block at 180 °C for 6 h (N = 3).

As it was confirmed that heating an oil without the addition of water induces an increase in AV, we then evaluated the contribution of free fatty acids to this increase in AV. Free fatty acids contained in the heated oil were fluorescently labeled with the 9-anthryldiazomethane (ADAM) reagent and quantified by high-performance liquid chromatography fluorescence detection (HPLC-FLD). As a result, heating the oil for 6 h at 180 °C induced only a slight increase in the amount of free fatty acids (palmitic acid, stearic acid, oleic acid, linoleic acid, and linolenic acid) contained in the oil. This slight increase in free fatty acids may be due to the hydrolysis of triacylglycerols by water that remains slightly in the oil even after heating (80–300 ppm; data not shown).

We then calculated how this slight increase in free fatty acids affected AV. As such, “free fatty acid-derived AV” was calculated based on the above quantification of free fatty acids. The following formula was used (where 56.11 corresponds to the molecular weight of KOH (g/mol)):

$${\text{Free fatty acid - derived AV}} = {\text{free fatty acid concentration }}\left( {{\text{nmol}}/{\text{g oil}}} \right) \, \times { 56}.{11}/{1}0^{{6}}$$

Free fatty acid-derived AV before and after heating the canola oil was 0.020 and 0.023, respectively, demonstrating an increase of only 0.003. Meanwhile, as mentioned above, the actual AV (determined by titration) increased from 0.054 to 0.241, demonstrating an increase of 0.187. Hence, these results strongly suggest that carboxylic acids other than free fatty acids were formed during the heating of the oil, and these carboxylic acids were measured as AV.

### Identification of carboxylic acids other than free fatty acids that contributed to AV

**Figure 2:**
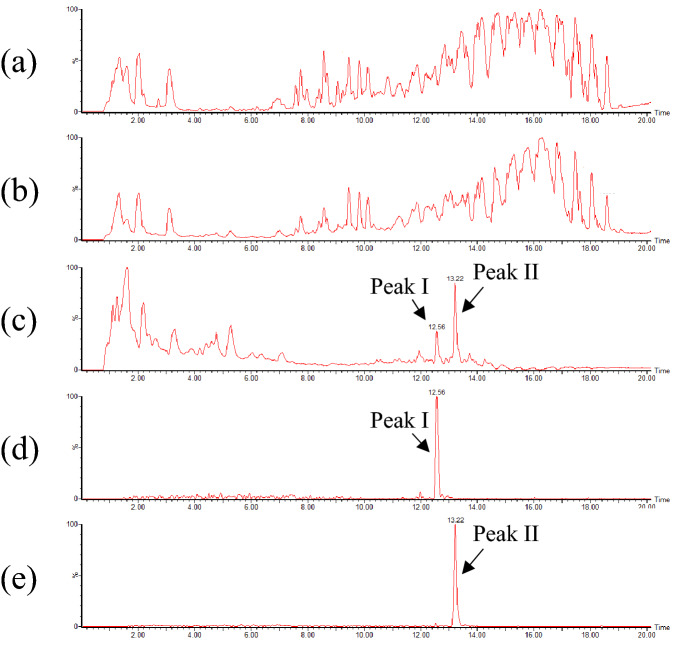
Typical chromatograms obtained during the UPLC-Tof/MS^E^ analysis of ADAM-derivatized heated canola oil. The heating of canola oil was performed at 180 °C for 6 h. MS chromatogram (**a**). Product ion chromatogram without selection of the precursor ion (**b**). Extracted ion chromatogram of the product ion chromatogram without selection of the precursor ion (**c**, at *m/z* 191.0861). Extracted ion chromatogram of the MS chromatogram (**d**, at *m/z* 1001.6832 [M + Na]^+^; and **e**, at *m/z* 1003.6985 [M + Na]^+^). Each peak is annotated with the retention time.

The above results suggested that carboxylic acids other than free fatty acids were formed during the heating of oils. We anticipated that these carboxylic acids would also react with the ADAM reagent, and their structures could be identified by analyzing the resultant ADAM derivatives. Hence, the above oil, heated for 6 h without addition of water, was derivatized with the ADAM reagent and analyzed with mass spectrometry. Collision induced dissociation of ADAM derivatives is known to yield a characteristic product ion of *m/z* 191, corresponding to an anthryl group^14–16^. Thus, we attempted to identify carboxylic acids in the ADAM-derivatized oil by searching for the precursor ions that afforded the fragment ion of *m/z* 191. This search was conducted using the UPLC-Tof/MS^E^ mode which simultaneously obtains the MS spectrum and product ion spectrum without selection of the precursor ion (i.e., data-independent MS/MS analysis; Fig. 2)^17–19^.

The extracted ion chromatogram of *m/z* 191.0861 (calcd. for C_15_H_11_, 191.0861; anthryl group) in the product ion chromatogram demonstrated two major peaks at the retention times of 12.56 min and 13.22 min (peak I and peak II; Fig. 2c). A comparison of these peaks with the MS chromatogram suggested that the precursor ion of each peak was *m/z* 1001.6832 ([M + Na]^+^ of peak I; calcd. for C_63_H_94_O_8_Na, 1001.6846; Fig. 2d) and *m/z* 1003.6985 ([M + Na]^+^ of peak II; calcd. for C_63_H_96_O_8_Na, 1003.7003; Fig. 2e), respectively. These peaks were hardly detected before heating (data not shown), suggesting that these ions were the ADAM derivatives of carboxylic acids formed during the heating of the oil. In addition to the above data-independent MS/MS analysis, product ion scan with the selection of precursor ions (*m/z* 1001.6832 and *m/z* 1003.6985) was conducted (Supplementary Fig. S1 online). The anthryl group-characteristic fragment ion (*m/z* 191) was observed in this product ion spectrum, confirming that m/z 1001.6832 and m/z 1003.6985 were ADAM derivatives.

**Figure 3:**
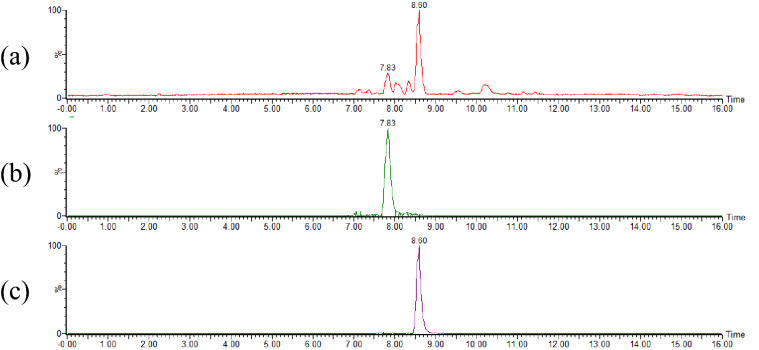
Typical chromatograms obtained during the UPLC-Tof/MS analysis of heated canola oil (underivatized with ADAM). The heating of canola oil was performed at 180 °C for 6 h. Base peak ion chromatogram (**a**). Extracted ion chromatogram (**b**, at *m/z* 787.6088; **c**, at *m/z* 789.6248). Each peak is annotated with the retention time.

Under the assumption that *m/z* 1001.6832 and *m/z* 1003.6985 each possess only one anthryl group (C_15_H_11_) in their structures, we considered that the molecular formula of each ion before ADAM derivatization was C_48_H_84_O_8_ (calcd. mass 788.6166 Da) and C_48_H_86_O_8_ (calcd. mass 790.6323 Da), respectively. Hence, to confirm that these carboxylic acids were indeed contained in the heated oil, oils that were not derivatized with the ADAM reagent were analyzed by UPLC-Tof/MS. As expected, the ions corresponding to these carboxylic acids, *m/z* 787.6088 ([M−H]^−^, calcd. for C_48_H_83_O_8_, 787.6088; Fig. 3a,b) and *m/z* 789.6248 ([M−H]^−^, calcd. for C_48_H_85_O_8_, 789.6244; Fig. 3a,c) were observed in the total and extracted ion MS chromatograms.

**Figure 4:**
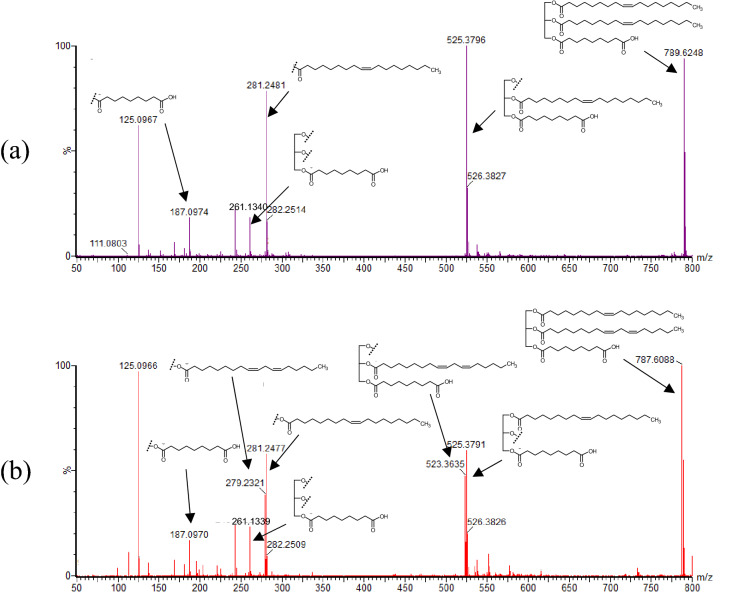
Product ion mass spectra of the target carboxylic acids. Heated canola oil (without ADAM derivatization) was analyzed by UPLC-Tof/MS. *m/z* 789.6 was selected as a precursor ion for (**a**) and *m/z* 787.6 was selected as a precursor ion for (**b**). The structures illustrated in this figure are the triacylglycerols bearing nonanedioic (azelaic) acid at the α position.

To further analyze the structure of *m/z* 787.6088 and *m/z* 789.6248, we selected these ions as precursor ions and conducted a product ion scan. Product ion scan of *m/z* 789.6248 afforded *m/z* 281.2481 (calcd. for C_18_H_33_O_2_, 281.2481) corresponding to oleic acid (Fig. 4a). Additionally, *m/z* 525.3796 (calcd. for C_30_H_53_O_7_, 525.3791) corresponding to the loss of one oleic acid, and *m/z* 261.1340 (calcd. for C_12_H_21_O_6_, 261.1338) corresponding to the loss of two oleic acids were detected. Hence, *m/z* 789.6248 was suggested to consist of two oleic acids. Furthermore, based on the characteristic ion of *m/z* 187.0974 (calcd. for C_9_H_15_O_4_, 187.0970) and the structures of *m/z* 525.3796 and *m/z* 261.1340, *m/z* 789.6248 was suggested to be a glycerol ester of two oleic acids and one C_9_H_16_O_4_. Since C_9_H_16_O_4_ must possess a free carboxyl group that can react with the ADAM reagent, we assumed that it was nonanedioic (azelaic) acid, a saturated 9-carbon dicarboxylic acid. Similarly, the product ion scan of *m/z* 787.6088 afforded product ions corresponding to oleic acid, linoleic acid, and nonanedioic acid (Fig. 4b). From these results, we identified 8-carboxy-octanoyl (azelaoyl)-triacylglycerols, namely, dioleoyl-azelaoyl-glycerol (C_48_H_86_O_8_) and oleoyl-linoleoyl-azelaoyl-glycerol (C_48_H_84_O_8_), as the main carboxylic acids contained in the heated canola oil that were not free fatty acids.

**Figure 5:**
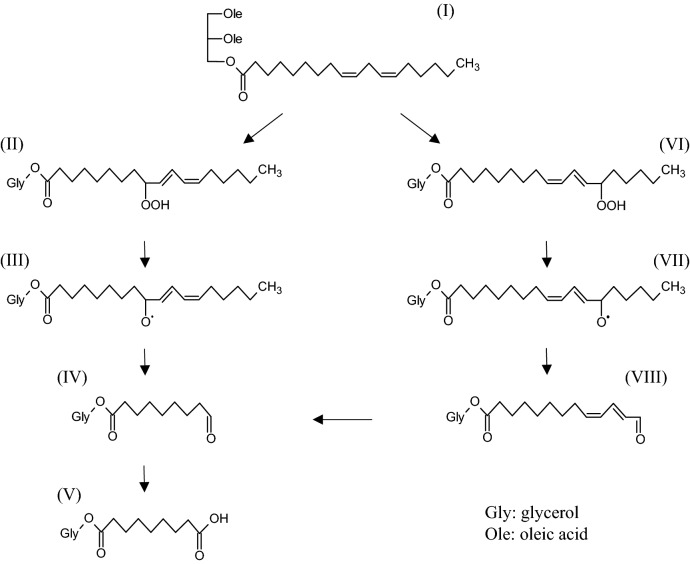
Proposed scheme of the formation of dioleoyl-azelaoyl-glycerol (V).

The fatty acid composition of the canola oil used in this study was 66.1% oleic acid and 19.1% linoleic acid (Supplementary Table S1 online). Hence, we assumed that triacylglycerols containing oleic or linoleic acid were thermally decomposed to afford azelaic acid. Based on the results of previous studies^20–25^ and this study, we propose the pathway described in Fig. 5. In the case of dioleoyl-linoleoyl-glycerol (I), its thermal oxidation affords dioleoyl-(9-hydroperoxyl-octadecadienoyl)-glycerol (II) or dioleoyl-(13-hydroperoxyl-octadecadienoyl)-glycerol (VI)^20–22^. After the formation of alkoxy radical III, a β-scission reaction between C9 and C10 of III yields dioleoyl-(9-oxo-nonanoyl)-glycerol (IV)^21, 23, 24^. Further oxidation of IV yields a C9 dicarboxylic compound (V)^25^. Similarly, after the formation of alkoxy radical VII, a β-scission reaction between C13 and C14 of VII yields dioleoyl-(13-oxo-tridecadienoyl)-glycerol (VIII). VIII may also be converted to IV^23^. Although C8 and C12 dicarboxylic acids can theoretically be formed through the β-scission of III and VII, the C9 dicarboxylic acid was mainly detected in this study, thus, other pathways may also be relevant.

### Quantification of azelaoyl-triacylglycerols in the heated oil

We confirmed above that the heated canola oil contained dioleoyl-azelaoyl-glycerol and oleoyl-linoleoyl-azelaoyl-glycerol. To quantify these azelaoyl-triacylglycerols, the heated oil was subjected to methyl esterification, and dimethyl nonanedioate (azelate), the expected product, was analyzed by GC-MS. As a result, dimethyl azelate was clearly detected from the heated oil (Supplementary Fig. S2 online). Other dimethyl dicarboxylates were either detected only in trace amounts or were not detected. The concentration of dimethyl azelate increased significantly from 1.4×10^2^ to 1.9×10^3^ nmol/g by heating the oil for 6 h.

We then calculated how this significant increase in azelaoyl-triacylglycerols affected AV. Hence, “azelaoyl-triacylglycerols-derived AV” was calculated based on the above quantification of dimethyl azelate. Azelaoyl-triacylglycerols-derived AV was calculated using the following formula:

$${\text{Azelaoyl - triacylglycerols - derived AV}} = {\text{dimethyl azelate concentration }}\left( {{\text{nmol}}/{\text{g oil}}} \right) \, \times { 56}.{11}/{1}0^{{6}} .$$

Azelaoyl-triacylglycerols-derived AV before and after heating the oil for 6 h was 0.008 and 0.109, respectively, demonstrating an increase of 0.101. Considering that the actual AV increased from 0.054 to 0.241 (an increase of 0.187), the increase in azelaoyl-triacylglycerols-derived AV (0.101) accounted for 54.0% of the increase in the actual AV. Meanwhile, as mentioned above, free fatty acid-derived AV increased by only 0.003 (corresponding to 1.6% of the increase in actual AV). Hence, the contribution of azelaoyl-triacylglycerols was considerably larger than that of free fatty acids. To the best of our knowledge, this is the first study to confirm that a compound other than a free fatty acid contributes to AV.

### Azelaoyl-triacylglycerols and their contribution to AV in an oil used to deep-fry potatoes

The water content of oils is known to increase during the cooking of foods (e.g., deep-fry) as the water contained in foods are transferred to the oil^26^. When an oil is heated in the presence of such water, triacylglycerols hydrolyze into free fatty acids, leading to an increase in AV. Many studies in fact have demonstrated that the AV of oils increase when oils are used to cook food (e.g., deep-fry potatoes)^11, 13, 27–31^. In addition to such an increase in free fatty acids, the results of the current study suggested that azelaoyl-triacylglycerols may also contribute to AV. Hence, we evaluated the contribution of azelaoyl-triacylglycerols to the AV of an oil used to deep-fry potatoes (i.e., an oil with a relatively high water content).

As such, a deep-frying test using frozen French fries was conducted. The total heating time of the oil was 270 min and the total frying time was 126 min. During frying, the average oil temperature was about 170 °C, and the water content was about 1200–2500 ppm. The AV before and after the test, determined by titration, was 0.054 and 0.284, respectively. This increase was comparable to that observed in previous studies where oils were used to cook foods^11, 13, 28–30^. Based on the quantification of free fatty acids and dimethyl dicarboxylates, free fatty acid-derived AV and azelaoyl-triacylglycerols-derived AV were calculated. As a result of deep-frying French fries, the free fatty acid-derived AV increased from 0.020 to 0.058, demonstrating an increase of 0.038. Meanwhile, azelaoyl-triacylglycerols-derived AV increased from 0.008 to 0.078, demonstrating an increase of 0.070. Considering that the actual AV (determined by titration) increased by 0.230 (from 0.054 to 0.284), free fatty acids and azelaoyl-triacylglycerols accounted for 16.5% and 30.4% of the increase in AV, respectively. These results indicate that the formation of azelaoyl-triacylglycerols can also occur during the cooking of foods (i.e., oils with a relatively high water content), and both free fatty acids and azelaoyl-triacylglycerols contribute to AV. Therefore, AV is not only an indicator of free fatty acids formed by the hydrolysis of triacylglycerols (as defined in ISO 660) but also an indicator of azelaoyl-triacylglycerols formed via the oxidation of triacylglycerols. Moreover, the progress of oil deterioration may be evaluated with higher accuracy by measuring free fatty acids and azelaoyl-triacylglycerols in addition to AV.

## Conclusion

This study identified azelaoyl-triacylglycerols as compounds that contribute to AV. Azelaoyl-triacylglycerols were found to contribute to the AV of an oil that was heated without addition of water and an oil that was used to cook French fries. Although AV, by definition, is an indicator of triacylglycerol hydrolysis, the results of the current study suggest that AV is also an indicator of azelaoyl-triacylglycerols formed by the oxidation of triacylglycerols. Further research on AV, based on these perspectives, should lead to a reduction in food loss by extending the life cycle of frying oils.

## Materials and methods

### Materials

Canola oil was manufactured by J-OIL MILLS, Inc. (Tokyo, Japan; Supplementary Table S1). Aquamicron AX and Aquamicron CXU were purchased from Mitsubishi Chemical Corporation (Tokyo, Japan). ADAM reagent was obtained from Funakoshi Co., Ltd. (Tokyo, Japan). Oleic acid, dimethyl nonanedioate, and methyl heptadecanoate were purchased from FUJIFILM Wako Pure Chemical Corporation (Osaka, Japan). Undecanoic acid was purchased from Tokyo Chemical Industry Co., Ltd. (Tokyo, Japan). Azelaic acid was obtained from Sigma-Aldrich (St. Louis, U.S.A.). Leucine-enkephalin was obtained from Waters (Milford, MA, U.S.A.). Frozen French fries were purchased from a market in Kanagawa, Japan. Other reagents were of the highest grade available.

### Analysis of canola oil heated without the addition of water

Fresh canola oil (10 g) was placed in a 51 mm stainless steel dish and heated on a digital heat block (Dry Thermo Unit DTU-2C, TAITEC, Tokyo, Japan) at 180 °C for 6 h. AV was measured according to the official method of the American Oil Chemists’ Society (AOCS, Cd 3d-63)^32^. Moisture content in the oil was measured by Karl Fischer titration with Aquamicron reagents using a CA-310 Moisture meter (Mitsubishi Chemical Analytech, Tokyo, Japan). The color of the oil was measured using a Lovibond PFXi-880/L (The Tintometer Limited, Amesbury, England) according to the official method of the AOCS (Cc 13e-92)^33^. Viscosity was measured by placing an oil sample (1.2 mL) between the cone and plate of a VISCOMETER TV-25 (Toki Sangyo, Tokyo, Japan). The measurement was started at 30 °C, and data was recorded every 30 seconds until 2 min. The average data was used as the viscosity.

Free fatty acids were analyzed by ADAM derivatization. Quantification was performed according to previous studies^14, 34, 35^ and the manufacturer's instructions as follows. Oil (200 mg) and undecanoic acid (internal standard, 0.3 mg) were dissolved in 10 mL of acetone. ADAM reagent (1 mg/mL in acetone, 100 µL) was added to 50 µL of this acetone solution, and the mixture was allowed to react for 16 h at room temperature in the dark. After the reaction, the solution was diluted 10-fold with acetone. The reaction mixture (5 µL) was analyzed by HPLC-FLD using an LC-20 series HPLC system equipped with a fluorescence detector (FLD-20A, Shimadzu, Kyoto, Japan). Separation was carried out on a Lichrosorb RP-8 column (4.0 mm I.D., 250 mm, 5.0 um, Merck, Darmstadt, Germany) at 40 °C. The flow rate of the mobile phase (A, water; B, acetonitrile) was set to 1.0 mL/min. The gradient was as follows: 60% of mobile phase B for 15 min, 60–90% of mobile phase B between 15 and 30 min. The excitation and emission wavelengths were set at 365 nm and 412 nm, respectively. A calibration curve was prepared using the area ratio between oleic acid and the internal standard^35^. The calibration curve was used to quantitate the concentration of each free fatty acid (palmitic acid, stearic acid, oleic acid, linoleic acid, and linolenic acid).

### Analysis of carboxylic acids other than free fatty acids contained in the heated canola oil

Carboxylic acids produced during the heating of canola oil were identified with UPLC-Tof/MS using an ACQUITY UPLC H-class system equipped with a Zevo G2-S qTOF/MS (Waters, Milford, MA, U.S.A.). Heated canola oil was derivatized with the ADAM reagent as described above. To search for candidate compounds containing the anthryl group, the reaction mixture (1 μL) was analyzed using the UPLC-Tof/MS^E^ mode (Condition 1 in Supplementary Table S2). The MS spectrum and product ion spectrum were obtained simultaneously without the selection of the precursor ion (i.e., data-independent MS/MS analysis)^17–19^. Product ion scan analysis with the selection of precursor ions was performed using Condition 2 described in Supplementary Table S2. Next, to identify the chemical structures of the candidate compounds, the heated canola oil, without ADAM derivatization, was analyzed by UPLC-Tof/MS. Heated canola oil was diluted 1000-fold with acetone, and 1 μL of the diluted sample was analyzed. The MS spectrum (Condition 3 in Supplementary Table S2) and the product ion spectrum with the selection of a precursor ion (Condition 4 in Supplementary Table S2) were obtained.

UPLC separations were performed using a CORTECS C18 column (2.1 mm I.D., 100 mm, 1.6 μm, Waters, Milford, MA, U.S.A.) at 55 °C. The flow rate of the mobile phase (A, methanol/water (1:1, v/v) containing 0.1% formic acid and 10 mM ammonium acetate; B, 2-propanol containing 0.1% formic acid and 10 mM ammonium acetate) was set to 0.2 mL/min. The gradient was as follows: 40% to 100% of mobile phase B between 0 and 15 min, 100% of mobile phase B between 15 and 20 min. MS parameters were optimized using the MassLynx v4.1 software (Waters, Milford, MA, USA). Leucine-enkephalin was used as the M.W. standard in the LockSpray mode. These systems provide a resolution of > 30,000 (full width at half maximum). The mass extraction window was set to ± 5 mDa. Elemental compositions were predicted based on accurate masses using the MassLynx v4.1 software.

### Quantification of azelaoyl-triacylglycerols

GC-MS (Agilent 7890A gas chromatograph coupled with an Agilent 5975C MS system, Agilent, Little Falls, DE, USA) was used to determine the total amount of azelaoyl-triacylglycerols. Azelaoyl-triacylglycerols were methyl-esterified according to the official method of the AOCS (Ce 2-66)^36^ and quantified in the form of dimethyl azelate. The hexane layer containing fatty acid methyl esters was diluted 10-fold with hexane and analyzed by GC-MS. Methyl heptadecanoate was used as an internal standard. GC separation was performed using a DB-WAX GC column (0.25 mm I.D., 60 m, 0.25 μm film thickness, GL Science, Tokyo, Japan). The GC oven was programmed as follows: the initial oven temperature was 40 °C for 5 min, increased to 190 °C at 3 °C/min and held for 5 min, and then increased to 240 °C at 10 °C/min and held for 30 min. The helium flow rate was kept constant at 1.2 mL/min. The electron ionization mode and the scan monitor mode were used to analyze dimethyl dicarboxylates. The peaks were identified with reference to previous studies^25^ with some modifications. Extracted ion chromatograms at *m/z* 152 and 143 were used to analyze dimethyl azelate and methyl heptadecanoate, respectively. A calibration curve was constructed based on peak area ratios (dimethyl azelate/internal standard) and applied to calculate the concentration of dimethyl azelate.

### Deep-frying test

A stainless steel pan was filled with fresh canola oil (600 g) and heated to 180 °C. Frozen French fries (100 g) were deep-fried at 180 °C for 7 min starting at 10:00 am. The fries were removed, and after an interval of 8 min, the next frozen fries were fried at 180 °C for 7 min. This process was repeated until six groups of French fries were fried. The heating of the oil was stopped at 11:30 am. The same oil was used to fry French fries in the same manner on the next day and the day after.

## Supplementary Information

Supplementary Information.

## Data Availability

The datasets used and/or analyzed during the current study available from the corresponding author on reasonable request.
