# Supplementary material for: Carboxylic acids derived from triacylglycerols that contribute to the increase in acid value during the thermal oxidation of oils
Source: Sci Rep. 2022 Jul 21;12:12460. doi: 10.1038/s41598-022-15627-3 (PMC9304340; doi:10.1038/s41598-022-15627-3)
Supplement: Supplementary file 1 — Supplementary Information. [file 41598_2022_15627_MOESM1_ESM.pptx]

## Slide 1
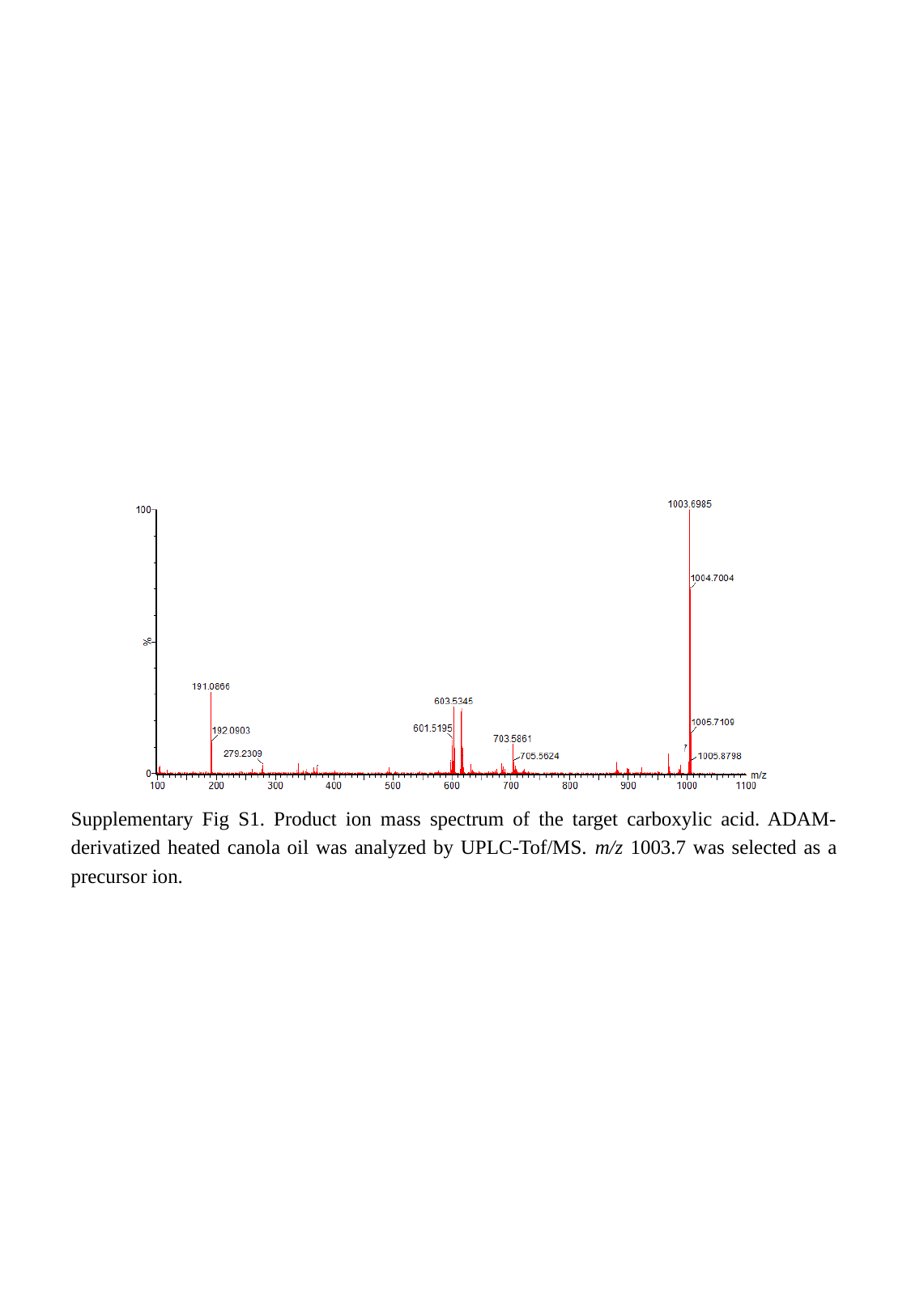

Supplementary Fig S1. Product ion mass spectrum of the target carboxylic acid. ADAM-derivatized heated canola oil was analyzed by UPLC-Tof/MS. m/z 1003.7 was selected as a precursor ion.

## Slide 2
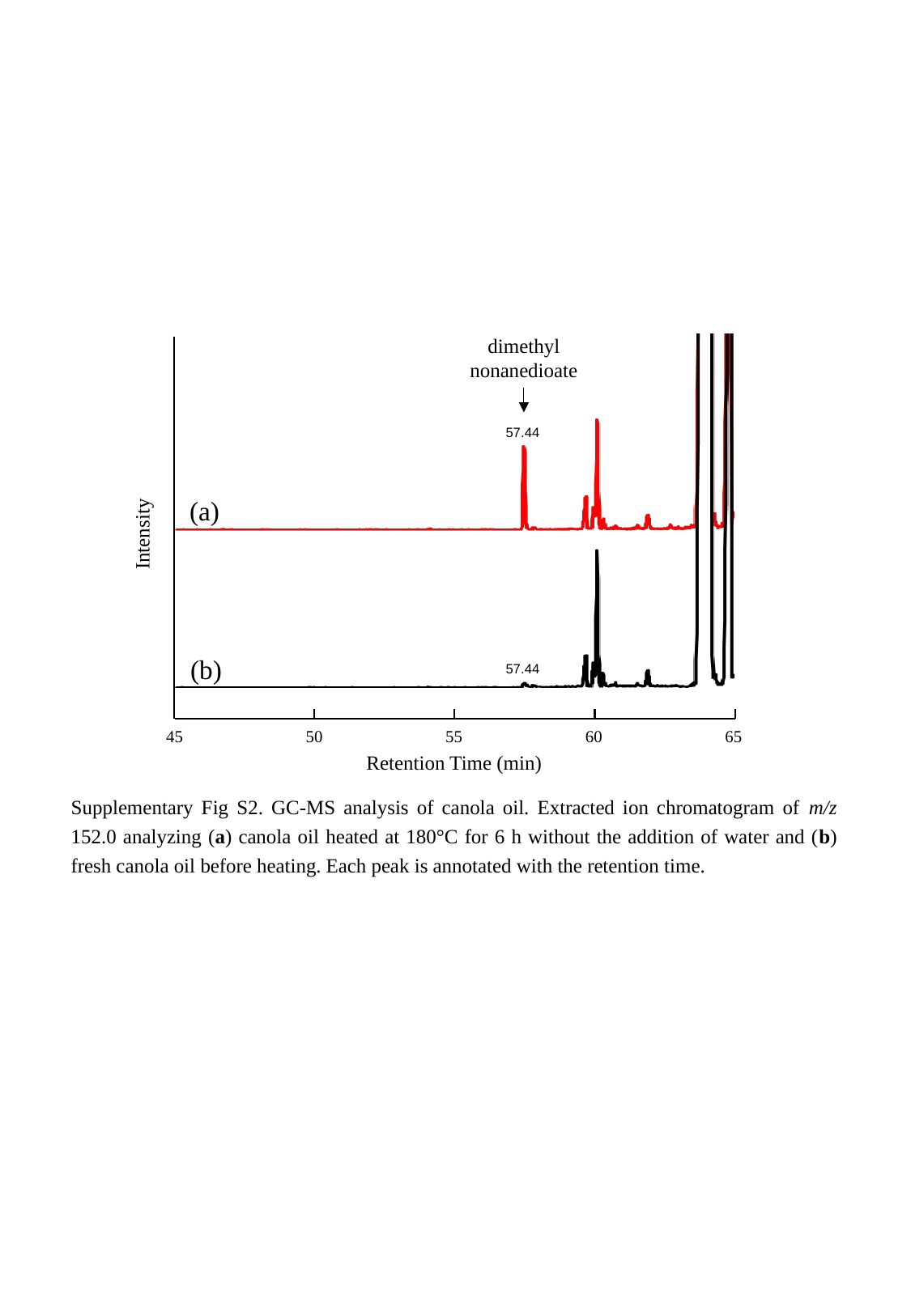

dimethyl nonanedioate
57.44
(a)
Intensity
(b)
57.44
45
50
55
60
65
Retention Time (min)
Supplementary Fig S2. GC-MS analysis of canola oil. Extracted ion chromatogram of m/z 152.0 analyzing (a) canola oil heated at 180°C for 6 h without the addition of water and (b) fresh canola oil before heating. Each peak is annotated with the retention time.

## Slide 3
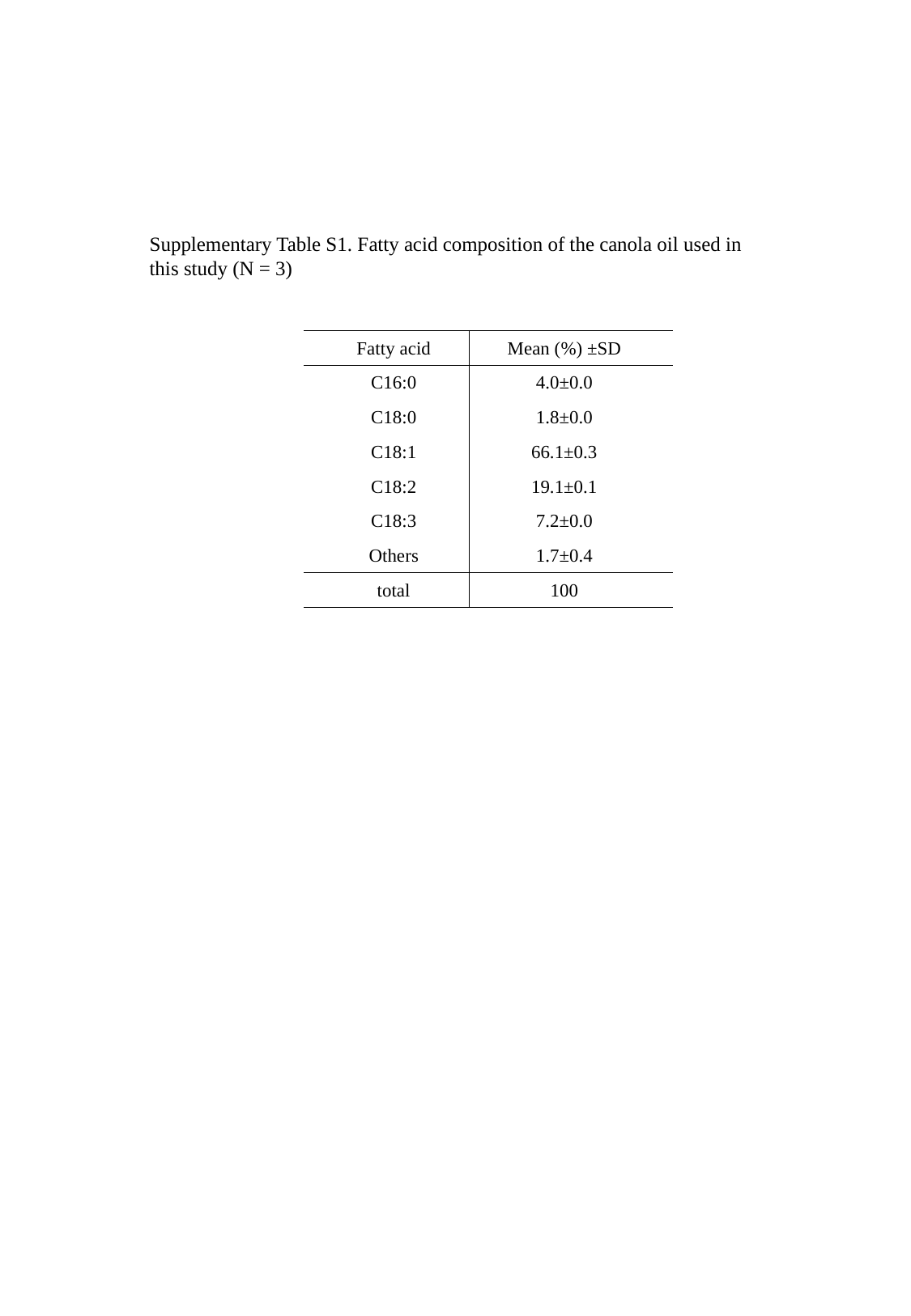

Supplementary Table S1. Fatty acid composition of the canola oil used in this study (N = 3)
| | Fatty acid | Mean (%) ±SD | |
| --- | --- | --- | --- |
| | C16:0 | 4.0±0.0 | |
| | C18:0 | 1.8±0.0 | |
| | C18:1 | 66.1±0.3 | |
| | C18:2 | 19.1±0.1 | |
| | C18:3 | 7.2±0.0 | |
| | Others | 1.7±0.4 | |
| | total | 100 | |

## Slide 4
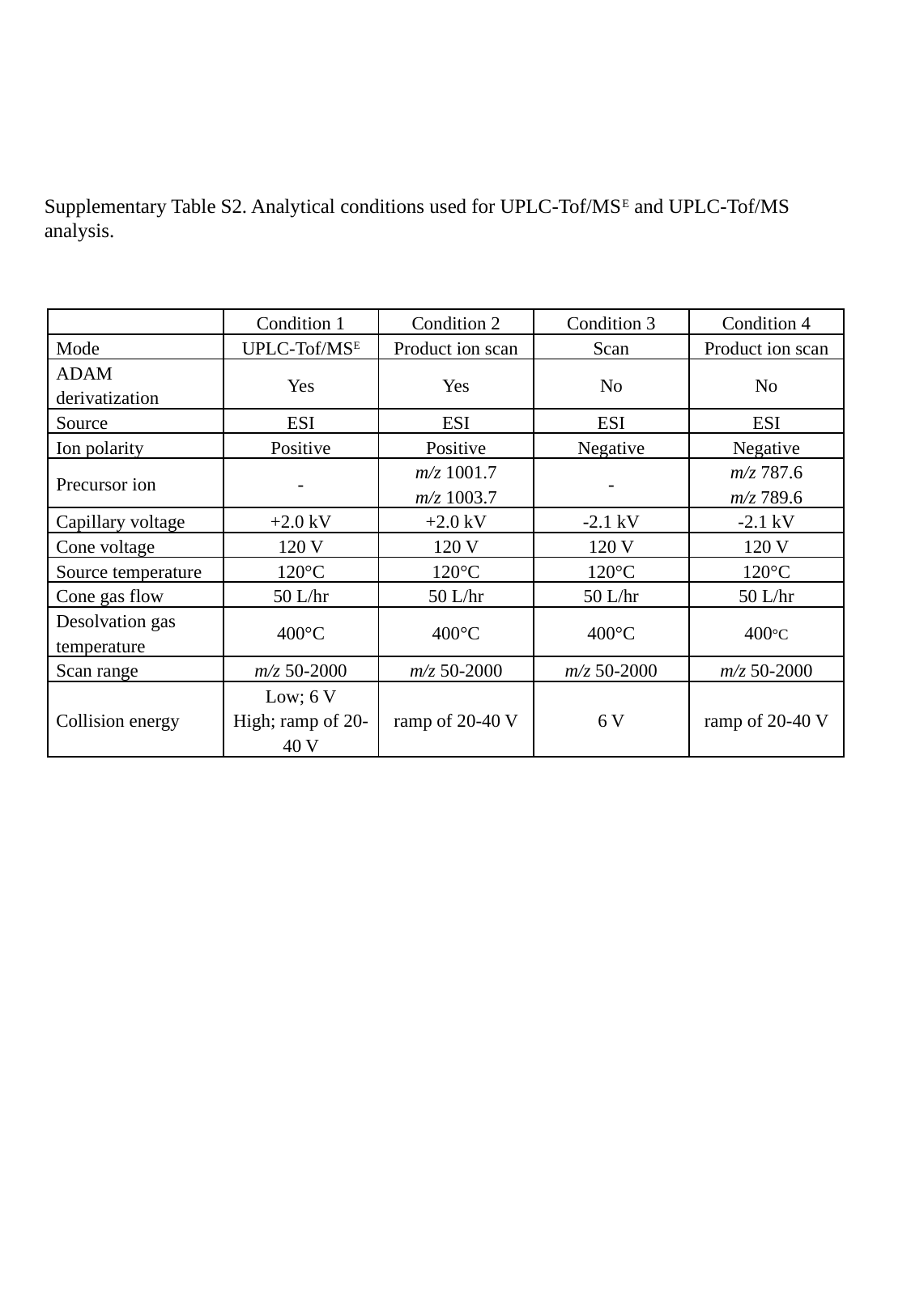

Supplementary Table S2. Analytical conditions used for UPLC-Tof/MSE and UPLC-Tof/MS analysis.
| | Condition 1 | Condition 2 | Condition 3 | Condition 4 |
| --- | --- | --- | --- | --- |
| Mode | UPLC-Tof/MSE | Product ion scan | Scan | Product ion scan |
| ADAM derivatization | Yes | Yes | No | No |
| Source | ESI | ESI | ESI | ESI |
| Ion polarity | Positive | Positive | Negative | Negative |
| Precursor ion | - | m/z 1001.7 m/z 1003.7 | - | m/z 787.6 m/z 789.6 |
| Capillary voltage | +2.0 kV | +2.0 kV | -2.1 kV | -2.1 kV |
| Cone voltage | 120 V | 120 V | 120 V | 120 V |
| Source temperature | 120°C | 120°C | 120°C | 120°C |
| Cone gas flow | 50 L/hr | 50 L/hr | 50 L/hr | 50 L/hr |
| Desolvation gas temperature | 400°C | 400°C | 400°C | 400°C |
| Scan range | m/z 50-2000 | m/z 50-2000 | m/z 50-2000 | m/z 50-2000 |
| Collision energy | Low; 6 V High; ramp of 20-40 V | ramp of 20-40 V | 6 V | ramp of 20-40 V |
